# Recent Advances in the Catalytic Hydroconversion of 5-Hydroxymethylfurfural to Valuable Diols

**DOI:** 10.3389/fchem.2022.925603

**Published:** 2022-06-03

**Authors:** Zexing Huang, Jianhua Wang, Jing Lei, Wenguang Zhao, Hao Chen, Yongjun Yang, Qiong Xu, Xianxiang Liu

**Affiliations:** ^1^ National and Local Joint Engineering Laboratory for New Petro-Chemical Materials and Fine Utilization of Resources, Key Laboratory of the Assembly and Application of Organic Functional Molecules of Hunan Province, Hunan Normal University, Changsha, China; ^2^ Chenzhou Gao Xin Material Co., Ltd., Chenzhou, China

**Keywords:** biomass, 5-hydroxymethylfurfural, hydroconversion, diols, catalysis

## Abstract

Biomass, a globally available resource, is a promising alternative feedstock for fossil fuels, especially considering the current energy crisis and pollution. Biomass-derived diols, such as 2,5-bis(hydroxymethyl)furan, 2,5-bis(hydroxymethyl)-tetrahydrofuran, and 1,6-hexanediol, are a significant class of monomers in the polyester industry. Therefore, the catalytic conversion of biomass to valuable diols has received extensive research attention in the field of biomass conversion and is a crucial factor in determining the development of the polyester industry. 5-Hydroxymethylfurfural (HMF) is an important biomass-derived compound with a C6-furanic framework. The hydroconversion of HMF into diols has the advantages of being simple to operate, inexpensive, environmentally friendly, safe, and reliable. Therefore, in the field of diol synthesis, this method is regarded as a promising approach with significant industrialization potential. This review summarizes recent advances in diol formation, discusses the roles of catalysts in the hydroconversion process, highlights the reaction mechanisms associated with the specificities of each active center, and provides an outlook on the challenges and opportunities associated with the research on biomass-derived diol synthesis.

## Introduction

Non-renewable fossil resources are pivotal for economic growth, and their consumption has surged steadily in recent decades (Zhou et al., 2011; Kucherov et al., 2018). Currently, more than 85% of the global energy demand is met using fossil fuel resources (Chen et al., 2018). Considering the probable impending energy crisis, the use of renewable resources and development of sustainable energy technology have been a focus area of research in recent years (Zhu et al., 2016; Wan et al., 2021). Traditional industries emit massive amounts of CO_2_ into the atmosphere, contributing to climate change and global warming (Ravishankara et al., 2015). Biomass conversion, which is a promising alternative to energy production using fossil hydrocarbons, has the potential to alleviate the energy crisis while effectively utilizing greenhouse gas emissions through completing the CO_2_ cycle (Nakagawa et al., 2013; Hu et al., 2018a; Deng et al., 2021).

5-Hydroxymethylfurfural (HMF), a widely used biomass platform chemical, has C=O, C-OH, and C=C bonds, all of which promote a variety of reactions. Reducing different types of unsaturated groups in HMF, various derivatives of HMF can be produced, such as 5-methylfurfural (MF) (Sun et al., 2019), 2,5-dimethylfuran (DMF) (Guo et al., 2020), 2,5-bis(hydroxymethyl)furan (BHMF) (Zhao et al., 2022), 2,5-bis(hydroxymethyl)-tetrahydrofuran (BHMTHF) (Kong et al., 2015), and 1,6-hexanediol (1,6-HD) (Yao et al., 2016). Selectivity is a crucial parameter in chemical reactions involving the major HMF derivatives utilized in a variety of industrial applications, including fuel production, polyurethane manufacturing, and polymer production (Chernyshev et al., 2017; Stadler et al., 2019; Li et al., 2021). Diol products derived from HMF have significant industrial value and development potential as key monomers for polyurethane and polyester manufacturers. Hence, the number of studies on HMF has been rapidly increasing.

Even though several reviews have summarized topics such as HMF oxidation/reduction and synthesis suitable for industrial production (Troiano et al., 2020; Yan et al., 2020), none of them have focused on introducing breakthroughs in research on biomass-derived diols. Hence, in this review, we summarize recent advances in the hydroconversion of HMF, emphasizing the formation of high-value diols *via* the selective hydrogenation of various functional groups on HMF, and analyze the synergistic effects of various catalyst components, to aid the advancement of biomass transformation research.

## Production of Diols With Ring Structures

This section summarizes recent advances in the catalytic hydrogenation of HMF to produce diols with ring structures, such as BHMF and BHMTHF. Diols formed through the hydrogenation of carbonyl inside HMF while retaining the ring structure are extremely useful in the synthesis of a variety of foams, polyethers, and crown ethers (Zhang et al., 2017; Kucherov et al., 2018). As a furan molecule, BHMF is more thermally and chemically stable than HMF (Zhu et al., 2021). Consequently, it is preferred as an industrial raw material for furan derivatives over HMF. Owing to the strong ring structure and symmetrical diol functional group, BHMF has unique advantages over conventional polyesters, especially for the production of linear or cross-linked polyurethane (Zhang et al., 2017). Additionally, BHMTHF should be more widely used than BHMF, as it has a low probability of forming by-products during polymerization (Stadler et al., 2019). For comparison, the experimental data are summarized in Table 1.

**TABLE 1:** Catalytic performance of catalysts for the conversion of HMF.

Entry	Catalyst	Hydrogen Donor	Solvent	T (°C)	*p* (MPa)	t (h)	Yield (%)	Ref
1^a^	Ru/MnCo_2_O_4_	H_2_	methanol	100	8.2	4.0	98.5	Mishra et al. (2020)
2^a^	Co@C	H_2_	methanol	110	3.0	6.0	96.0	Arias et al. (2020)
3^a^	meso-Cu/Al_2_O_3_	H_2_	Ethanol	70	5.0	3.5	98.6	Kim et al. (2021)
4^a^	imp-Cu/Al_2_O_3_	H_2_	Ethanol	70	5.0	3.5	72.3	Kim et al. (2021)
5^a^	cp-Cu/Al_2_O_3_	H_2_	Ethanol	70	5.0	3.5	93.0	Kim et al. (2021)
6^a^	Cu/Al_2_O_3_	H_2_	methanol	130	3.0	1.0	92.1	Rao et al. (2021)
7^a^	Ni_x_Co_y_	H_2_	THF	100	0.5	4.0	93.1	Zhao et al. (2021)
8^a^	Zr-DTPA	isopropanol	isopropanol	140	/	4.0	95.2	He et al. (2021)
9^a^	Zr-HTC	isopropanol	isopropanol	120	/	4.0	99.2	Hu et al. (2018a)
10^a^	MZCCP	isopropanol	isopropanol	140	/	2.0	93.4	Hu et al. (2019)
11^a^	ZrBa-SBA	isopropanol	isopropanol	150	/	2.5	90.6	Wei et al. (2018)
12^a^	Hf-LigS	isopropanol	isopropanol	100	/	2.0	90.0	Zhou et al. (2019)
13^a^	Zr-LigS	isopropanol	isopropanol	100	/	1.0	60.6	Zhou et al. (2019)
14^a^	CuO-Fe_3_O_4_/AC	Ethanol	Ethanol	150	/	5.0	92.4	Fan et al.(2019)
15^a^	ZrBa-SBA	isopropanol	isopropanol	150	/	2.5	90.6	Zhang et al. (2020)
16^b^	Ru/CeO_x_	H_2_	1-butanol/water	130	2.8	12.0	89.0	Alamillo et al. (2012)
17^b^	Ru (methylallyl)2COD	H_2_	toluene	120	1.0	16.0	87.0	Cadu et al. (2018)
18^b^	Ni-Al	H_2_	Dioxane	60	6	6	96.2	Kong et al. (2015)
19^b^	Ni-Co-Al	H_2_	methanol	120	4	4	89.0	Yao et al. (2016)

a yield to BHMF.

b yield to BHMTHF.

### Hydrogenation of Carbonyl Inside HMF to BHMF With H_2_

The majority of hydrogenation reactions use H_2_ as a hydrogen donor because of the high BHMF yield obtained at relatively mild reaction temperatures in hydrogen environments (Gilkey et al., 2016). Ru/MnCo_2_O_4_ displayed high catalytic activity in methanol at a temperature of 100°C and a pressure of 8.2 MPa H_2_ for 4 h (Mishra et al., 2020). The high yield of BHMF (98.5%) was due to the high dissociation capacity of Ru, Brønsted acidity of the MnCo_2_O_4_ spinel support surface, and increase in the Lewis acidity induced by ruthenium nanoparticles. In comparison to noble metals, transition metals have lower costs and adequate reserves, and consequently, they have attracted considerable attention from researchers. As a result, Co, which is a transition metal, has been widely used for the catalytic conversion of HMF to BHMF. A hydrothermally synthesized monodispersed metallic Co catalyst, Co@C, was used in the selective hydrogenation of HMF, resulting in a 96.0% yield of BHMF at 110°C and 1 MPa H_2_ for 6 h with methanol as the solvent (Arias et al., 2020). According to this study, the excellent performance of the Co@C catalyst is attributable to the minuscule Co^0^ nanoparticles coated on the surface of the carbon shell, which provide sufficient catalytic activity.

**FIGURE 1:**
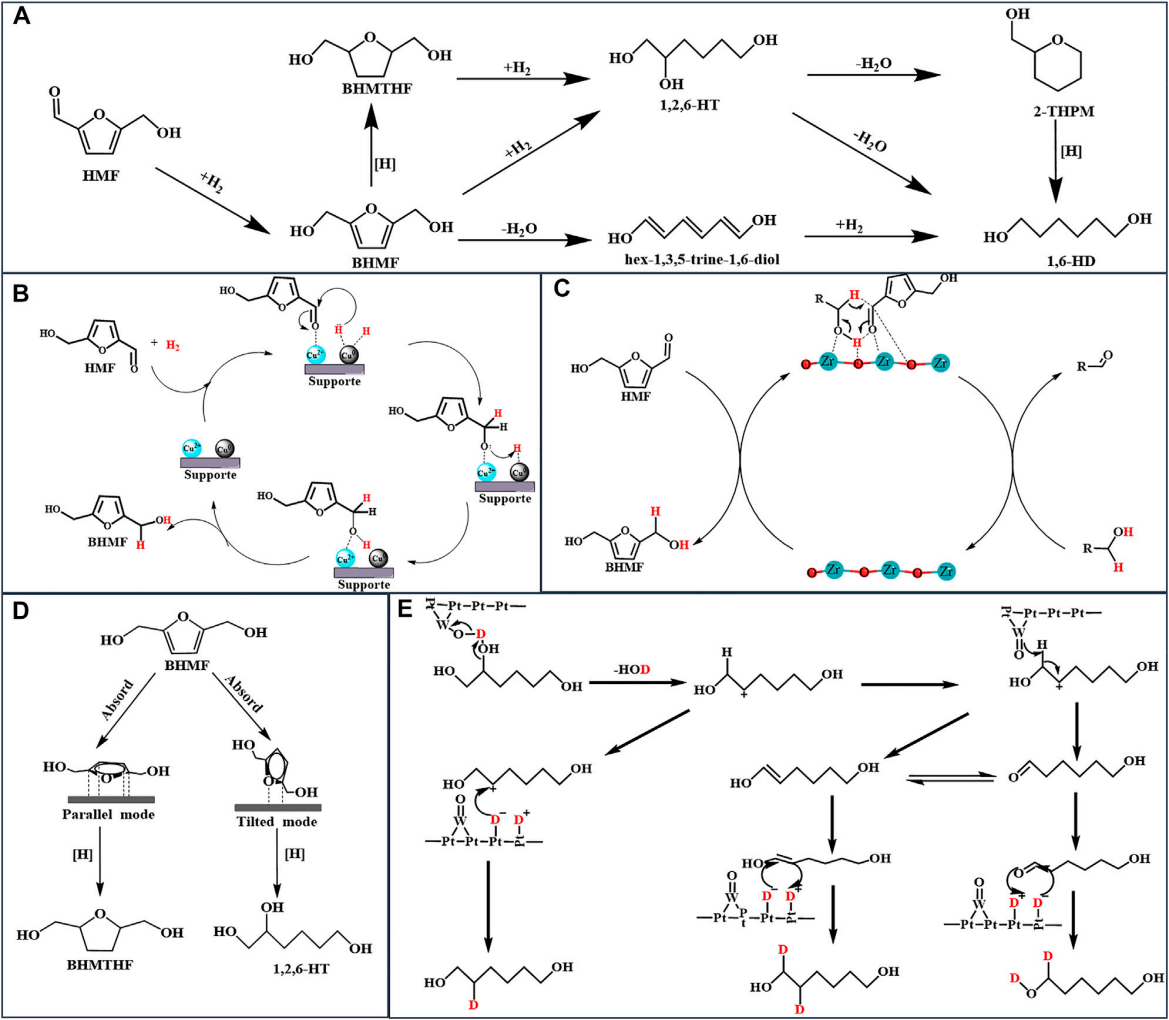
**(A)**: The route of formation of 1,6-HT from HMF; **(B)**: Mechanism for the selective conversion of HMF to BHMF over the Cu/Al_2_O_3_ catalyst **(C)**: Mechanism for the CTH of HMF to BHMF over the Zr-based catalyst **(D)**: Adsorption configurations of BHMF **(E)**: Mechanism for the selective cleavage of C-OH over the Pt-WO_*x*_/TiO_2_ catalyst.

As previously mentioned, smaller metal nanoparticles have higher dispersion and a more number of catalytically active sites (Arias et al., 2020). Hence, the regulation of the size of the active metal center is critical when designing metal catalysts. Kim’s work provides in-depth guidelines regarding the same for researchers. Kim developed structurally different copper-alumina catalysts, including meso-Cu/Al_2_O_3_, cp-Cu/Al_2_O_3_, and imp-Cu/Al_2_O_3_, via solvent-deficient precipitation (SDP), coprecipitation (CP), and impregnation (IMP) methods, respectively, and used them to catalyze the hydrogenation of HMF (Kim et al., 2021). Under the same reaction conditions, as shown in Table 1, entries 4, 5, and 6, the meso-Cu/Al_2_O_3_ catalyst outperformed both impregnated and co-precipitated Cu/Al_2_O_3_ catalysts in terms of catalytic activity. This phenomenon is attributable to the fact that SDP approaches provide catalysts with a higher BET surface area, which is 1.5 and 2 times larger than that obtained via cp-Cu/Al_2_O_3_ and imp-Cu/Al_2_O_3_, respectively; Additionally, it provides a Cu surface area more than double of that achieved using other methods, especially for meso-Cu/Al_2_O_3_. The SDP method, which utilizes NH_4_HCO_3_ as the precipitator rather than NaHCO_3_, transforms Cu^2+^ in the solvent into a fine and uniformly distributed solid crystal, resulting in better dispersion than other catalysts. Along with the size of the metal nanoparticles, the proportion of different active centers significantly influences the catalytic effect. Rao and co-workers described the mechanism of hydrogenation of HMF by Cu/Al_2_O_3_ in detail. As illustrated in Figure 1B, the coexistence of Cu^0^ and Cu^2+^ species on the catalyst surface affects the catalytic performance (Rao et al., 2021). First, the presence of electrophilic Cu^2+^ aids the adsorption and activation of the C=O group of HMF via the oxygen lone pair. Hydrogen is dissociated on the Cu^0^, and the activated carbonyl group on Cu^0^ is then hydrogenated, resulting in the production of BHMF. Notably, different synthetic methods always result in diverse catalytic performance and physical properties of the catalysts.

Under certain reaction conditions, monometallic catalysts cannot simultaneously provide high conversion and selectivity. However, bimetallic systems can outperform monometallic catalysts in terms of catalytic activity owing to the synergistic effects induced by the geometric and electronic interactions of both metals involved (Elsayed et al., 2020; Zhang et al., 2020). Zhao and co-workers used a nickel–cobalt bimetallic catalyst to catalyze the hydrogenation reaction of HMF, obtaining a yield of 93.1% BHMF in a tetrahydrofuran (THF) solvent at 0.5 MPa hydrogen pressure and a reaction temperature of 100°C for 4 h (Zhao et al., 2021). An analysis of the effect on the Ni:Co ratio revealed that Ni exhibited strong catalytic reduction activity, resulting in C-O bond cleavage for the production of DMF, whereas Co inhibited the hydrogenolysis reaction, allowing BHMF to be retained and increasing BHMF selectivity. According to kinetic study results, the active energy required for HMF to BHMF conversion, 39.98 kJ mol^−1^, is significantly lower than that required for MF, 62.64 kJ mol^−1^, whereas the active energy required by BHMF for further transformation, 76.25 kJ mol^−1^, is higher than that of MF, 44.21 kJ mol^−1^, which is the crucial reason for the high yield of BHMF. Notably, the general reason for catalyst performance degradation after the repeated usage is that the substances absorbed on the catalyst surface during reaction warp the active sides, and this issue can be resolved through calcination in a suitable atmosphere (Huo et al., 2021; Zhao et al., 2022).

### Meerwein-Ponndorf-Verley Reaction of HMF to BHMF

While H_2_ as a hydrogen donor has the advantage of a low reaction temperature, it also has a number of disadvantages, including storage risk, transportation constraints, and minimal safety (Hu et al., 2018b). In comparison to H_2_, alcohol has the advantages of being easy to transport, having a large supply, and being completely safe. Therefore, researchers have focused on the use of various alcohols as hydrogen donors to convert HMF into BHMF via the Meerwein-Ponndorf-Verley (MPV) reaction. Recently, Elsayed et al. reported the use of a magnetic catalyst, CuO-Fe_3_O_4_/AC, to selectively hydrogenate HMF via catalytic transfer hydrogenation (CTH). Although CuO-Fe_3_O_4_/AC catalyzed the conversion of HMF to BHMF efficiently at 150°C with a 92% yield of BHMF, the reduction of Cu^2+^ by ethanol hinders the reusability of the catalyst (Elsayed et al., 2020). Hence, the development of highly efficient and stable catalysts is crucial for the formation of BHMF from HMF via the CTH strategy.

To accomplish the formation of BHMF *via* CTH, zirconium is commonly used as an active center in the design of a catalyst because Zr^2+^ has a strong adsorption capacity and electronegativity, which cause the substrate to form a six-membered ring with the alcohol, utilizing the Lewis base synergistically to complete the MPV reaction (Hu et al., 2018a). Figure 1C illustrates the related steps in detail. Although feasible, the CTH of HMF necessitates a high reaction temperature to achieve high conversion using a common supported catalyst. Enhancing the Lewis acid-base property of the catalyst to improve catalytic activity can efficiently reduce the required temperature. Hu and co-workers synthesized a series of high-performance zirconium-containing organic-inorganic nanohybrid catalysts with isopropanol as a clean hydrogen donor (Table 1, entries 8–10) (Hu et al., 2018a; Hu et al., 2019; He et al., 2021). In comparison to the supported catalyst (Alamillo et al., 2012; Wei et al., 2018), Table 1, entries 12 and 13, these metal ligand catalysts efficiently catalyzed the conversion of HMF into BHMF at a mild reaction temperature of 120–140°C with a superior BHMF yield of 93.4–99.2%. Their excellent CTH capacity and stability are due to the Lewis acid-base coordination of organo-functional groups with zirconium (He et al., 2021). Zhou and co-workers evaluated a series of inorganic-biopolymer hybrids (Hf, Zr, Fe, Al, and Zn-Ligs) for catalytic performance to CTH of HMF at 100°C with isopropanol as the hydrogen donor, and the obtained results indicated that only Hf-Ligs and Zr-Ligs showed BHMF selectivity (Zhou et al., 2019). The use of Hf-Ligs as the catalyst resulted in a 90.0% yield of BHMF, which was significantly higher than the yield of Zr-Ligs (60.6%). As Hf-Ligs have the highest base/acid molar ratio among the reported Zr-based catalysts, the CTH reaction temperature is reduced to 100°C.

Many studies have revealed that secondary alcohols are more active than primary alcohols owing to their lower reduction potential, which implies that they can more easily supply hydrogen and promote the CTH of HMF (Fan et al., 2021). There are two shortcomings while considering alcohol as a hydrogen donor in the literature reported: 1) Temperatures required for the reaction are usually high because the active energy required for converting HMF to BHMF *via* CTH is nearly twice as much as that required while using H_2_, and 2) when compared to the alcohol oxidized into the corresponding aldehyde or ketone, H_2_ as a hydrogen donor has the benefit of producing no by-products.

### Reduction of the Furan Ring Inside HMF to BHMTHF

For the synthesis of BHMTHF, Ru-based catalysts are typically utilized. Cadu pointed that carbene ligands offered moderate activity and selectivity, with a preference for unsaturated backbones and for bulky aromatic substitution pattern. Di-phosphorus containing ligands offered the highest conversion, leading to a good isolated yield of a mixture of BHMTHF, 87.0% (Cadu et al., 2018). Under optimal reaction conditions, the catalytic conversion of HMF to BHMTHF by Ru/CeO_x_ yields 89.0% with slight ring-opening by-products, 1,2,6-hexanetriol (1,2,6-HT) and 1,2,5-hexanetriol (1,2,5-HT) (Alamillo et al., 2012). In addition, the non-noble metal based catalysts has been applied in synthesis BHMTHF. Kong synthesized Ni-Al hydrotalcite-like catalyst and used it in formation of BHMTHF. The Ni-Al reduced at 450°C exhibited excellent performance to catalytic hydrogenation of HMF into BHMTHF, 96.2% yield under 6Mpa at 60°C for 6 h (Kong et al., 2015).

The above literatures indicated that preventing C-O cleavage is the key for high selectivity of BHMTHF. Yao and co-workers demonstrated that HMF can be converted directly to 1,2,6-HT by opening the furan ring without converting it first to BHMTHF (Yao et al., 2016). The ring-opening reaction did not occur in the upper layer below 100°C but accelerated rapidly as the reaction temperature increased to 120°C. Increasing the temperature resulted in a significant decrease in the 1,2,6-HT yield (41.9–20.1%), but no significant change in the BHMTHF yield (14.6–14.5%) was observed, indicating the attainment of an equilibrium between the amount of BHMTHF generation and consumption. Yang and co-workers prepared a Pd supported on layered double hydroxides (LDHs) catalyst for the selective hydrogenation of aromatic furfurals into aliphatic tetrahydrofurfural derivatives with over 92.0% selectivity and achieving a close-to-complete conversion of HMF (Yang et al., 2020). The effects on the solvent suggest that the polar solvent can prevent LDHs from adsorbing carbonyls, resulting in excellent selectivity for tetrahydrofurfural derivatives. As shown in Figure 1D, different HMF adsorption configurations on metal surfaces result in different product distributions. The C-O-C of BHMF activated by the catalyst was strongly adsorbed on the catalyst surface and then cleaved by reactive hydrogen. Correspondingly, the catalyst triggered the parallel adsorption of the furan ring of BHMTHF and then reduced it to BHMTHF. The geometric selectivity of the two absorption modes is due to the structure of LDHs, which prevents BHMF from moving. Notably, LDH catalysts exhibit the ability for parallel adsorption to the furan ring (Yao et al., 2016; Yang et al., 2020; Kong et al., 2015).

## Straight Chain Diol Production

Apart from the catalytic hydrogenation of the carbonyl group on the furan ring, the furan ring undergoes a variety of diverse and complex reactions, as illustrated in Figure 1A (Buntara et al., 2011; Yao et al., 2016; Enjamuri et al., 2020). Among those products, 1,6-HD is the key monomer of polyesters, polyester polyols, and polyurethanes (Krishna et al., 2017; Li et al., 2016; Zhang et al., 2017). Owing to its low environmental impact and high performance, 1,6-HD is widely used, primarily in polyurethanes. Because of the high requirements for downstream products, such as thermoplastic polyurethanes, elastomers, and coatings, the demand for 1,6-HD is increasing rapidly. Excellent properties, such as a high mechanical strength, low glass transition temperature, and high heat resistance, lead to unique advantages compared to other polyurethanes. Despite the fact that the overall output of 1,6-HD reached over 15.65 t/a in 2018, the global 1,6-HD market continues to grow at a rapid pace owing to the rising polyurethane demand (Enjamuri et al., 2020).

Buntara and co-workers demonstrated for the first time that 1,6-HD can be synthesized using HMF via a multi-step process that comprises the hydrogenation of the furan ring to BHMTHF, followed by the cleavage of the C-O-C bond to 1,2,6-HT and 1,6-HD (Buntara et al., 2011). According to a 2018 study, the Pt-WO_*x*_/TiO_2_ catalyst catalyzes the cleavage of the C-O-C bond inside BHMTHF to form 1,2,6-HT, which is then converted into 1,6-HD (He et al., 2018). The H atoms (H^+^ and e^−^) produced through hydrogen dissociation diffuse across TiO_2_, during which electrons reduce Ti^4+^ to Ti^3+^ and then react with a W=O species, reducing W^6+^ to W^5+^ to form a Brønsted acid site, W-OH, as illustrated in Figure 1E. Finally, the Brønsted acid site catalyzed C-O cleavage of BHMTHF and 1,2,6-HT to complete hydrodeoxidation. Stephens and co-workers reported a mechanistic study of the conversion of 1,2,6-HT to 1,6-HD over Pt-WO_*x*_/TiO. Using D_2_ and H_2_, the hydroxyl group on C2 was dehydrated, resulting in carbocation via W-OH species, and thereafter, W-O- acquired the hydrogen atom on C1 to form an aldehyde, which interacted with D_2_ to form 1,6-HD (Stephens et al., 2020). Noble metals have a high hydrogen dissociation capacity and reduce metal oxides to metal hydroxides or create oxygen vacancies to catalyze the dehydration of hydroxyl groups. As a result, the design of catalysts, with metal oxidation and noble metals, has emerged as a prominent strategy for the selective cleavage of C-OH (Guan et al., 2020; Stephens et al., 2020).

Furthermore, there is an efficient strategy for the direct conversion of HMF into 1,6-HD utilizing double-layered catalysts of Pd/SiO_2_ + Ir–ReO_*x*_/SiO_2_ in a fixed-bed reactor (Xiao et al., 2016). The bottom layer Pd/SiO_2_ catalyst was used for the hydrogenation of the furan ring and carbonyl to BHMTHF, and Ir–ReO_*x*_/SiO_2_ was used for the hydrodeoxygenation of BHMTHF to 1,6-HD. When THF was replaced with water, the yield of 1,6-HD increased, but the yield of BHMTHF decreased. The yield of 1,6-HD decreased as the amount of water increased owing to the formation of hexanol during hydrogenolysis. Water acts as a polar solvent, causing the oxonium to escape. The hydrogenolysis of 1,2,6-HT is a challenging step in 1,6-HD synthesis. Therefore, it is necessary to investigate novel reaction pathways. For the first time, the Pd/ZrP catalyst was employed to directly synthesize 1,6-HD from HMF at atmospheric pressure using formic acid (FA) (Tuteja et al., 2014). The dissociation of FA distributed on the catalyst, formation of *in situ* hydrogen, and adsorption to C-O-C and C=O bonds via electrostatic interactions with the acidic ZrP support contributed to this outcome. Mechanistic studies indicated that HMF was adsorbed onto the active center and that the support of the catalyst enabled the direct conversion of the furan ring into hex-1,3,5-trine-1,6-diol under the action of acidic sites. With three conjugated C=C bonds, the intermediate reacted with H_2_ derived from FA *via* Pd to form 1,6-HD. Despite new literature and research into 1,6-HD, converting HMF into 1,6-HD with a high yield *via* a one-step process remains challenging with the existing technology.

## Conclusions and Perspectives

Biomass has received significant interest in recent decades as a renewable feedstock. Highly efficient catalysts play an important role in selective biomass conversion. In this respect, metal catalysts have been extensively used in the hydrogenation of HMF with promising results. To synthesize BHMF, it is necessary to selectively reduce the C=O bond rather than cleave the C-H bond. The generation of nonaromatic diols from HMF should be the focus of future research. Notably, the polyester industry has a high demand for 1,6-HD, which is synthesized *via* the ring-opening reaction of BHMF or BHMTHF and reduction of the hydroxyl of 1,2,6-HT (1,6-HEXANEDIOL MARKET, 2019; GLOBAL 1,5-PENTANEDIOL MARKET, 2019). For a number of reasons, the formation of diols is difficult: 1) Despite prior research demonstrating the feasibility of using nickel- and cobalt-based catalysts for the synthesis of BHMTHF from HMF, the reduction or ring-opening of the furan ring requires a high H_2_ pressure; 2) the hydroxyl group has a low reactivity without the conjugation of the furan ring. These are the challenges that hinder the development of diol production.

Considering the current state of development, some prospects for the advancement of diol production are identified as follows: 1) Using a liquid hydrogen donor to convert HMF to biomass-derived diols employing a non-noble metal catalyst seems promising owing to the abundance of biomass, ease of transportation, and safety. FA as a hydrogen donor does not produce any additional by-products, and being a Brønsted acid, FA can assist in the ring-opening reaction of substances, allowing the formation of 1,6-HD efficiently. Although alcohol oxidation occurs while using the CTH strategy to form BHMF, the low-boiling-point ketone and aldehyde can be removed via purification. 2) The conversion of BHMF into a valuable derivative appears to be more promising than the conversion of HMF, which is neither thermally nor chemically stable. Hence, employing glucose or even cellulose as a raw material to directly synthesize BHMF with high selectivity seems advantageous. Zhang and co-workers achieved a 48.2% BHMF yield in a fixed bed reactor by directly converting fructose at 140°C over a Cu/ZnO/Al_2_O_3_ catalyst. The conversion of cellulose to BHMF might lead to significant advancements in biomass research. 3) The catalysis of the Brønsted acid, which forms up on metal oxidation after reaction with H atoms for the C-O bond, is essential for the cleavage of the C-OH and C-O-C bonds. Although noble metal catalysts can successfully catalyze the synthesis of 1,6-HD, their high costs render the diol production challenging. Therefore, developing a non-noble metal catalyst to catalyze the synthesis of 1,6-HD is a challenge that must be addressed. Employing a metal-ligand catalyst with a Brønsted acidic functional group could represent a breakthrough in the production of the 1,6-HD from HMF using a non-noble catalyst. Acidic functional groups act directly on the C-O bond, instead of metal oxidation. The direct formation of 1,6-HD from HMF, as the main monomer of the polymer, is expected to inspire researchers working in the field of biomass conversion.
